# Machine Learning augmented docking studies of aminothioureas at the SARS-CoV-2—ACE2 interface

**DOI:** 10.1371/journal.pone.0256834

**Published:** 2021-09-09

**Authors:** Monika Rola, Jakub Krassowski, Julita Górska, Anna Grobelna, Wojciech Płonka, Agata Paneth, Piotr Paneth

**Affiliations:** 1 Faculty of Chemistry, Lodz University of Technology, Lodz, Poland; 2 Center for Bioinformatics (ZBH), Universität Hamburg, Hamburg, Germany; 3 FQS Poland, Fujitsu, Kraków, Poland; 4 Department of Organic Chemistry, Faculty of Pharmacy, Medical University of Lublin, Lublin, Poland; 5 International Center for Research on Innovative Biobased Materials (ICRI-BioM) – International Research Agenda, Lodz University of Technology, Lodz, Poland; Kingston University, UNITED KINGDOM

## Abstract

The current pandemic outbreak clearly indicated the urgent need for tools allowing fast predictions of bioactivity of a large number of compounds, either available or at least synthesizable. In the computational chemistry toolbox, several such tools are available, with the main ones being docking and structure-activity relationship modeling either by classical linear QSAR or Machine Learning techniques. In this contribution, we focus on the comparison of the results obtained using different docking protocols on the example of the search for bioactivity of compounds containing N-N-C(S)-N scaffold at the S-protein of SARS-CoV-2 virus with ACE2 human receptor interface. Based on over 1800 structures in the training set we have predicted binding properties of the complete set of nearly 600000 structures from the same class using the Machine Learning Random Forest Regressor approach.

## Introduction

Subsequent outbreaks of pandemics [1] culminating in the current Covid-19 highlighted the necessity of protective actions from the scientific community. This was manifested in the initial attempts of repurposing currently used drugs, followed by a search for novel antiviral compounds and vaccines. Although the effort put into the studies of agents preventing infection caused by the SARS-CoV-2 virus worldwide is impressive, neither new effective drugs have been discovered nor there is a reassurance that vaccines will catch up with the fast mutations of the virus. This indicates the need for the evaluation of the antiviral activity of synthesizable compounds. In the case of current pandemics the search started with docking approach, pioneered by the most extensive study [2] of the inhibition at the interface between the spike protein (S-protein) of the virus and the human ACE2 receptor, responsible for the viral recognition of host cells (see reference [3] for a recent summary). These studies, while quite exhaustive, were restricted to about 9000 compounds although performed with the aid of one of the fastest available supercomputers.

In the chemoinformatics toolbox for studies of ligands interaction with enzymes, the reliability of methods diminishes from molecular dynamics to docking to various variants of Quantitative Structure-Activity Relationship (QSAR). However, the rate of processing ligand structures increases dramatically in the same order. Thus different QSAR methods should allow the exploration of large sets of potential antiviral compounds. The main drawback in applying this approach lies in the fact that it requires large data sets on the activity of closely related compounds to build reliable models. Such data is usually missing, especially when the need for models is urgent. In the lieu of experimental data, the results of docking might be used, although one has to keep in mind that the results of docking do not always correlate with bioactivity.

We have previously [4] attempted building a QSAR model of interactions between the series of compounds containing linear or cyclic N-N-C(S)-N structural motif with the interface between the virus S-protein and the ACE2 receptor but a training set of only 160 compounds did not lead to a reliable QSAR model. In this contribution, therefore, we have extended the number of considered ligands over 10-fold (to 1820) by the inclusion of compounds that can be readily synthesized. We have carried out docking studies using four different docking algorithms and subsequently used these docking scores as the training set and subsequently predicted binding properties of the complete library of 597780 structures from the same class by applying fingerprint-based Machine Learning model employing Random Forests classifiers [5–7] which rate outperforms classical QSAR methods by a few orders of magnitudes.

We have selected compounds with the NH-NH-C(S)-NH motif because it already got significant attention in medicinal chemistry. Biological activities of thiosemicarbazides, the simplest hydrazine derivatives of thiocarbamic acid, are considered to be related to their ability to form chelates with zinc, iron, nickel, copper, and other transition metal cations that play an important role in biological processes.[8] As a result, thiosemicarbazide ligands in their nitrogen and sulfur (N, S) bidentate form or (N, N, S) tridentate form are considered interesting targets for drug design and variety of bioactive compounds with potent antibacterial, antifungal, anticancer, anti-HIV, antiviral, insecticidal, antisclerotic, antioxidant, radical scavenging, and antiparasitic activity are reported in the literature every year.[9, 10] These sulfur and nitrogen donor ligands have attracted singular *attention due to* their inhibitory activity against the smallpox virus and protozoa influenza as well.[11] Some industrially important applications like the regulation of plant growth, anticorrosion activity, and antifouling effects have also been reported for these derivatives.[12, 13] Due to NH-NH-C(S)-NH structural motif, 1,4-disubstituted thiosemicarbazides are convenient precursors for the synthesis of their heterocyclic analogs with 1,2,4-triazole or 1,3,4-thiadiazole cores. Antibacterial, antifungal, antituberculosis, antimalarial, antileishmanial, antiviral, antioxidant, anticancer, antidiabetic, antihypertensive, diuretic, neuroprotectant[4, 14–20] activities were reported for these compounds. They *have* also well-documented *activity* as *CNS* depressants, cannabinoid CB1 receptor antagonists, PDE4A inhibitors, γ-aminobutyric acid-A (GABA-A) α-2, α-3 and α-5 containing receptor antagonists, analgesic, anticonvulsant, anti-inflammatory, and analgesic agents.[21–24] Additionally, many drugs containing 1,3,4 thiadiazole moiety such as acetazolamide, methazolamide, andmegazol or 1,2,4-triazole ring such as fluconazole, itraconazole, posaconazole, voriconazole, ravuconazole, estazolam, alprazolam, etizolam, rizatriptan, trapidil, trazodone, anastrozole, letrozole, ribavirin, and loreclezole are available in clinical therapy.

## Results

The main focus of the present studies was on the identification of efficient evaluation of bioactivity of compounds containing NH-NH-C(S)-NH motif, which was based on the ability of their binding to the S-protein of SARS-CoV-2 virus—ACE2 human receptor interface which structure was retrieved from the Protein Data Bank (PDB: 6M0J [25]). Considered substituted structures of thiosemicarbazides, thiadiazoles, and triazoles are schematically presented in Fig 4 while all obtained results of docking are collected in Table S1 deposited in the public repository (see Data Availability section). The studied molecules included linear carbonylthiosemicarbazide skeleton and its three cyclic derivatives: 1,3,4-thiadiazole, and 1,2,4-triazole (in the thiol and thionic forms) cores decorated by five different five-member rings as the C-substituent and substituted phenyl ring as the N-substituent. In total 1820 structures including all mono-, di-, and di*ortho*-*para*-halogen- substituted R^2^ substituents have been used.

Four docking scoring functions have been used. These include Vina (Windows implementation in the Chimera environment), FlexX and Hyde (implemented in LeadIT), and ChemPLP (implemented in Gold)—see Materials and Methods section for details. Note that ChemPLP scores, in contrast to the other algorithms employed herein, use mathematical formulas in which the more favorable interactions result in a higher score.

**Fig 1:**
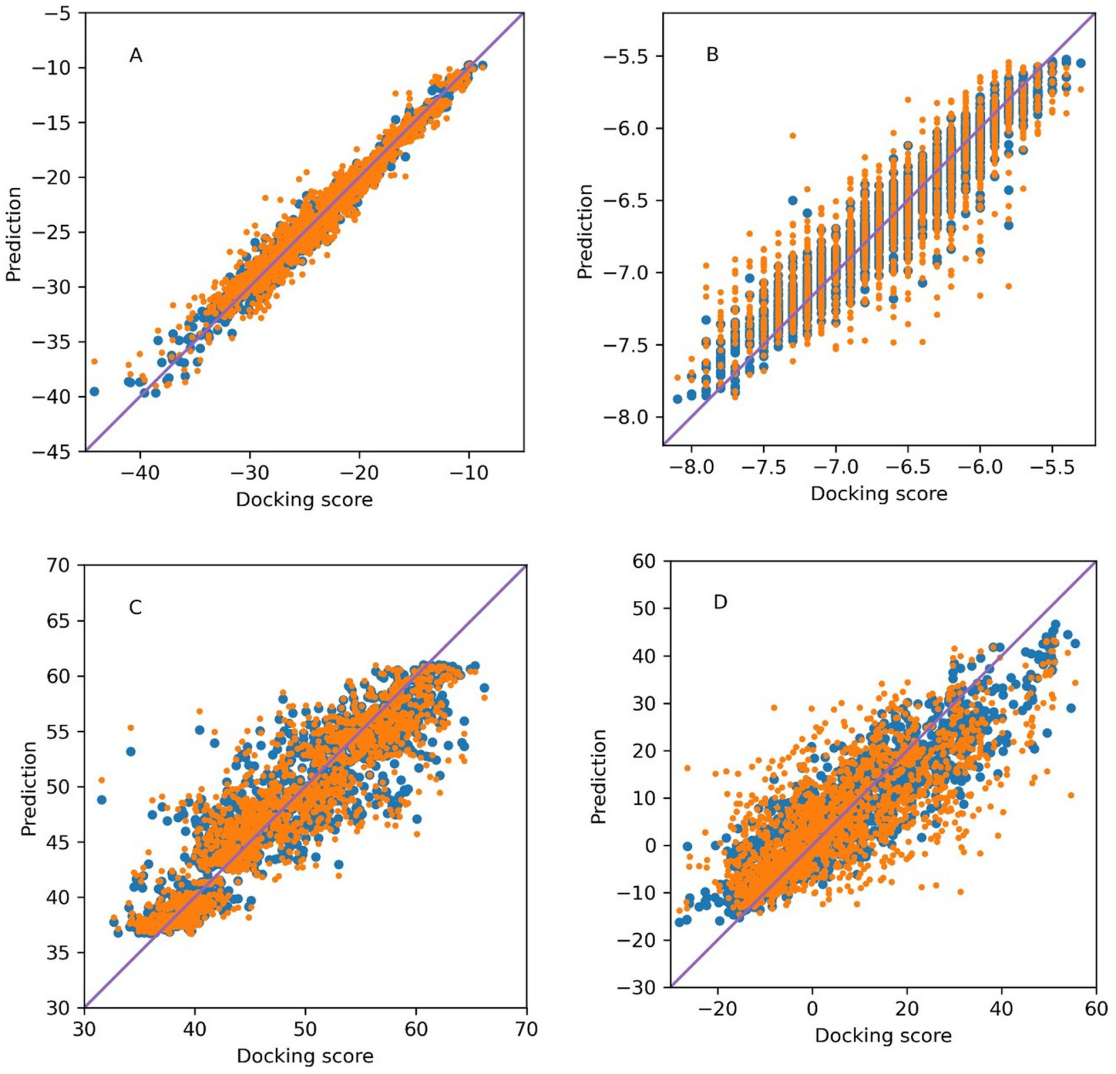
Predicted vs original docking scores obtained by Random Forests for scores computed by FlexX (A), Vina (B), ChemPLP (C), and Hyde (D). Blue points—learning stage, orange points–leave one out cross-validation stage.

Subsequently, Machine Learning models using Random Forest Regressor have been trained on all four sets of docking results (see Materials and Methods). The correlations obtained during training (R^2) and leave one out validation (Q^2) quantifying models performance are summarized in Table 1 and illustrated in Fig 1. The values of Q^2 above 0.75 are generally an indication of a model with useful predictive capabilities. The best fit was obtained for FlexX, while Vina and ChemPLP docking yielded acceptable correlations. A somewhat worse correlation between the docking scores and molecular fingerprints has been obtained with Hyde.

**Table 1:** R^2 and Q^2 values for ML-QSAR Random Forests modeling of docking scores.

	FlexX	Vina	ChemPLP	Hyde
R^2	0.99	0.93	0.86	0.82
Q^2	0.96	0.82	0.82	0.57

## Discussion

**Fig 2:**
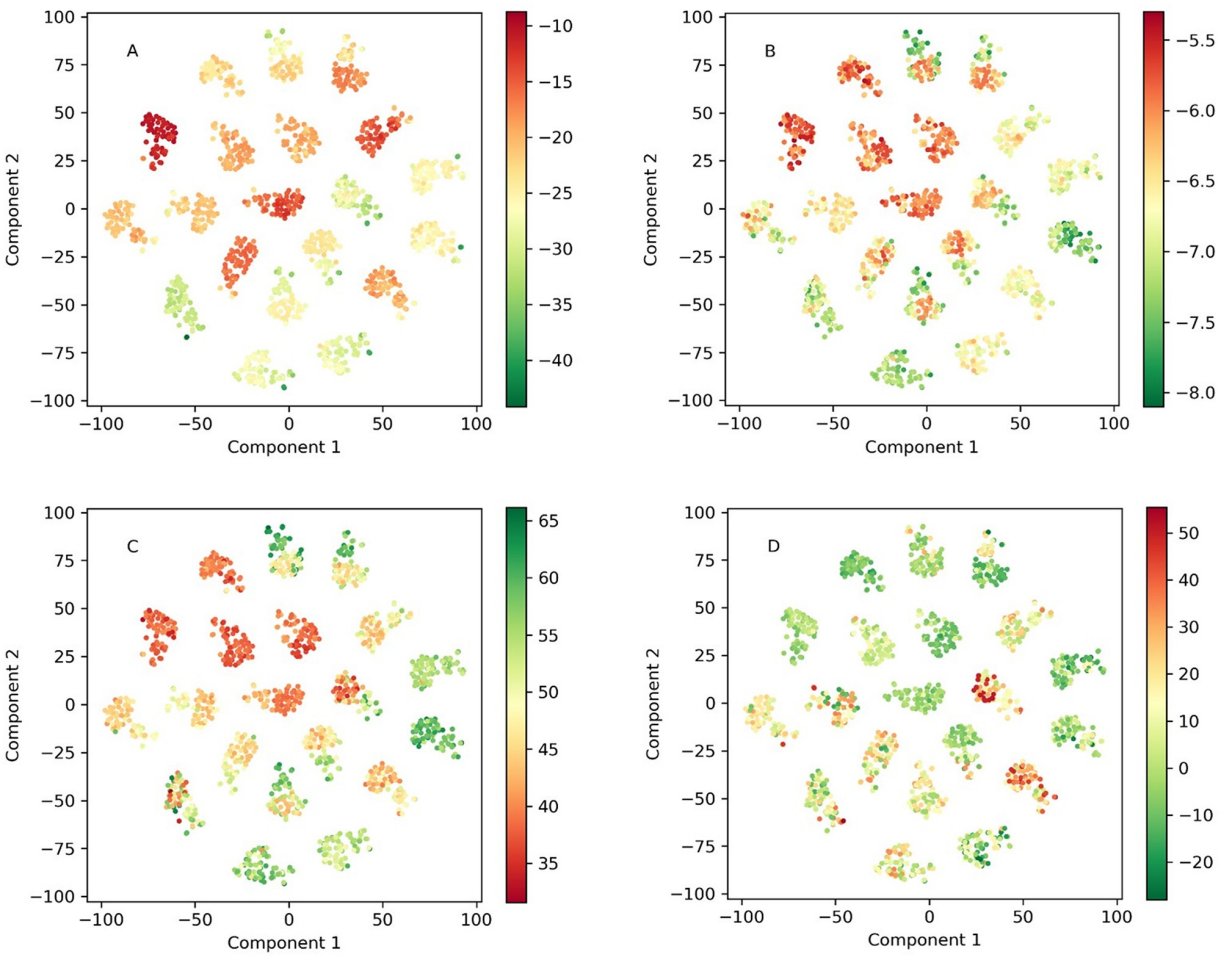
Two component t-SNE analysis of the set containing 1820 compounds in the 4096-dimensional space of Morgan Fingerprints colored by FlexX (A), Vina (B), Gold (C), and Hyde (D) docking scores.

Fingerprint-based Random Forests Regressors model yielded excellent correlation in the case of FlexX results, very good in cases of Vina and ChemPLP, and slightly worse in the case of Hyde. Since no methods of direct visualization of Random Forests exist we have deepened the analysis of the results by performing t-distributed Stochastic Neighbor Embedding (t-SNE) [26] analysis of the descriptor space, as it excels over methods like Principal Component Analysis for highly dimensional data [27]– 4096 dimensions in our case. This analysis is illustrated in Fig 2. The 20 clusters appearing in the t-SNE plots were verified to represent the significantly chemically different groups of compounds (all combinations of core moieties and R^1^ substituent). The ability of t-SNE to identify the chemically different groups of compounds confirms the choice of fingerprints to describe our compounds. It should be noted that the activity data of the compounds was not used in the t-SNE analysis, it was only added at the stage of plot preparation. Any correlations observed between the activity (presented as color in Fig 2) and the position of the molecule in the t-SNE plots should be interpreted as intrinsic correlations between the activity and chemistry of the molecule. A cluster represents a group of molecules with similar fingerprint patterns, that can be understood as a structural similarity. The appearance of clusters uniform in color (= activity), especially visible in Fig 2A suggests that small variations in structural features do not significantly contribute to the activity. While there is a consensus between docking scores of FlexX, Vina, and ChemPLP, Hyde scores appear to vary within each cluster, a fact that might explain the lower performance of the SAR modeling of Hyde scores.

As we have indicated, computational tools that can be used for the prediction of bioactivity of different compounds need nowadays to be very fast to be able to cope with big data collections. Machine Learning techniques like Random Forests outperform significantly other methods such as molecular dynamics, docking, and classical QSAR. While our previous studies [28, 29] involving similar techniques hinted at Random Forest performing much better than classical QSAR in the modeling of the docking scores we were unable to sufficiently support such a conclusion due to the limited number of compounds studied. Our present results provide clear evidence that Random Forests calculations trained on docking results can provide an improved scientific tool with better rate and precision of predictions that allow evaluation of properties of hundreds of thousands of compounds in a realistic time. The practice of training fast methods on more precise ones is in fact quite common in computational chemistry. For example, computationally cheaper molecular mechanics force fields can be trained on data from expensive high-level *ab initio* computations.

**Fig 3:**
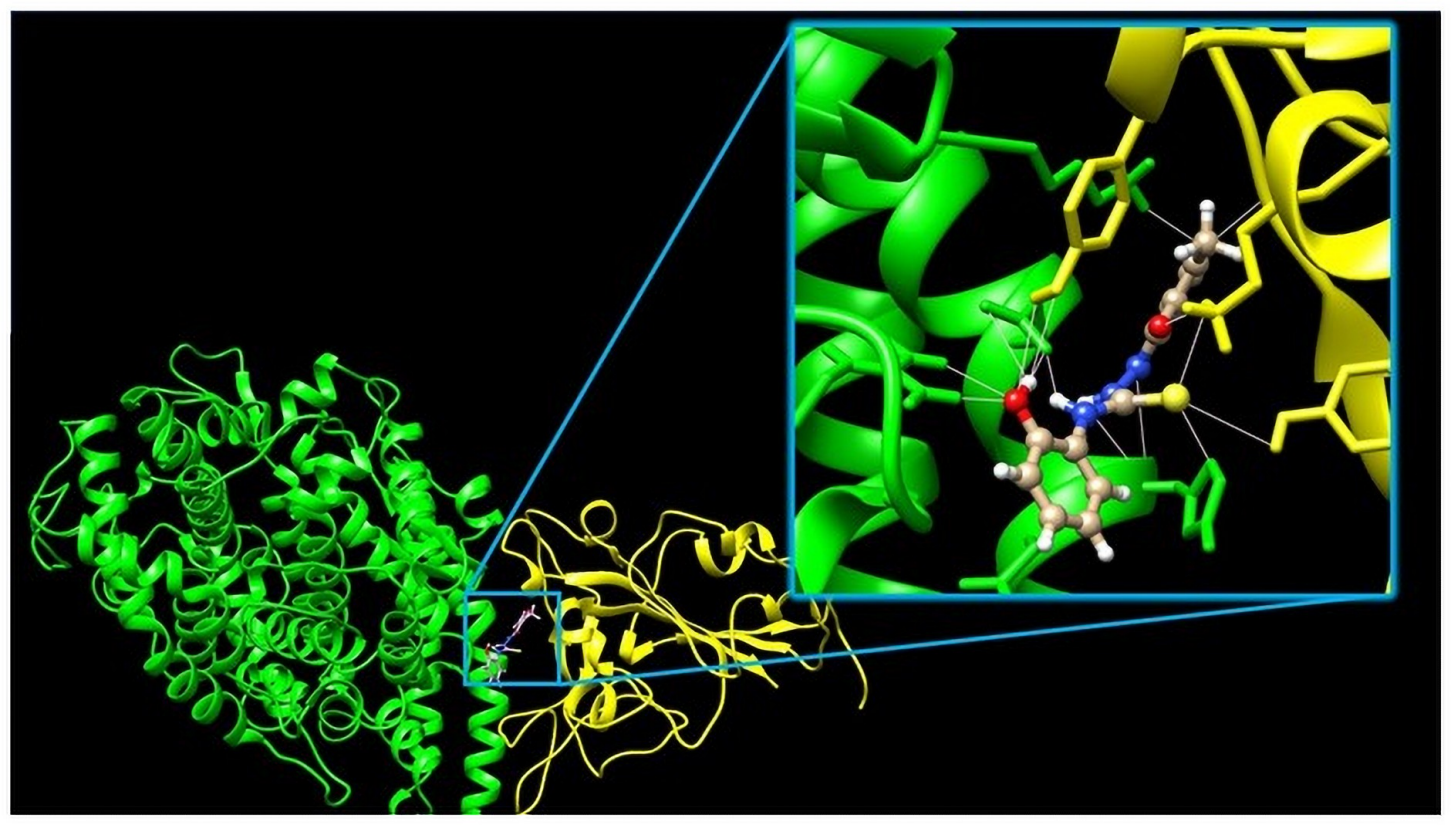
The orientation of the best result of the consensus docking (see the first line of Table 3) at the SARS-CoV-2 S-protein (yellow)—ACE2 receptor (green) interface. The insert shows the closest environment of the docked compound and the hydrogen bonding network.

However, having evaluated a large library of nearly 600000 compounds comprising the -N-N-C(S)-N- motif, we did not identify any compound that would be a better candidate for the lead compounds for further drug development than those which were in the training set. Therefore, below we discuss the results obtained from docking. Due to the lack of experimental data, and thus our inability to put more trust into particular docking algorithms used, we have ordered all results within a given docking protocol from the best to worst and assigned them a rank corresponding to the position on the list. In this way, the four best compounds have been identified. Subsequently, we have compiled a similar list according to the average rank in all four docking protocols, a “consensus” ranking [30]. These five best compounds are collected in Fig 5. In general, these results indicate that the linear thiosemicarbazides arrangement is preferred, these compounds occupy the first 20 positions on the consensus rank list. This result is not too surprising taking into account the length of the interface rim. Within the best-scored compounds, the majority contain the hydroxyl group in the *ortho* position of the R^2^ substituent. Compounds highly substituted in the phenyl ring did not score high, although triply substituted, with both *ortho* positions occupied scored highest in the case of ChemPLP and Vina docking. The interactions in the groove connecting S-protein (presented in yellow) with ACE2 receptor (presented in green) are illustrated in Fig 3 on the example of the molecule corresponding to the best result of the consensus docking presented in the first line of Fig 5. As indicated in the inset of Fig 3 the molecule is held rigidly by a network of hydrogen bonds (marked as pink lines) by both proteins. Sulfur atom forms hydrogen bonds with Tyr719, Lys669 on the spike protein side, and His16 of the human receptor. Also, the oxygen atom of the furan ring forms hydrogen bonds with both proteins; Gly762 of the spike protein and Lys335 of the ACE2 receptor. Hydroxyl group forms multiple hydrogen bonds with Agr375 and Glu19 of ACE2 and Tyr771 of the spike protein. Finally, both protons of the -NH-NH- fragment are in hydrogen bonding contact with His16 and Asp15 although the N-H…O angles are low indicating that these hydrogen bonds are very weak. In the blind docking to the SARS-CoV-2 S-protein–ACE2 receptor complex, as well as to both these proteins separately docking scores are best at the illustrated position indicating that its mode of action would be stabilization of the complex and thus trapping the virus rather than inhibiting its complexation with the receptor.

All compounds collected in Fig 5 were subjected to ADMET analysis. Major properties pertinent to selecting lead compounds [31] are collected in Table 2. As can be seen, they compare favorably with these of the two drugs tried clinically against Covid-19 (chlorquine and remdesivir).

**Table 2:** ADMET properties of best results of docking in comparison to clinically tried drugs.

Property	FlexX	Vina	ChemPLP	Hyde	consensus	chlorquine	remdesivir
Lipiński’s rules	+	+	+	+	+	+	-
solubility	soluble	moderate	moderate	soluble	soluble	poor	soluble
gastrointestinal absorption	high	high	high	high	high	high	low
acute toxicity (LD50)	2.576	2.589	2.549	2.496	2.385	2.642	2.990
Human hepatotoxicity (H-HT)	0.828	0.838	0.744	0.664	0.788	0.822	0.822
drug induced liver injury (DILI)	0.812	0.806	0.712	0.802	0.868	0.468	0.785
ames mutagenicity	0.402	0.368	0.328	0.386	0.374	0.810	0.270
hERG blockers	0.314	0.318	0.399	0.232	0.230	0.882	0.532

## Materials and methods

### Docking

Four docking algorithms were used. In the case of the FlexX algorithm [32], as implemented in the LeadIT platform [33], docking space was defined as a sphere with a radius of 7.5 Å centered at the point (83.5, 37.5, 110.0 Å) in the middle of the rim of the interface. Two different strategies were used. The first one was docking corresponding to a rigid receptor. In the second a 100 Å^3^ penetration of the van der Waals radii was allowed to account for the protein flexibility. The results of both docking strategies were found to be highly correlated and therefore only the results of docking with “flexible” protein receptor were considered in further studies. All best structures obtained for individual ligands using FlexX were subsequently subjected to docking refinement by a relatively new algorithm Hyde [34] implemented in the same platform. For docking using Vina [35] the standalone Windows-based executable has been used. The binding site was limited to the interface space by defining a 100.0x65.0x80.0 ÅxÅxÅ box centered at the same point as in FlexX docking using a visualization tool implemented in the Chimera [36] program. The same box was defined in studies using SwissDock [37] but it has been neglected by the server and blind docking has been performed instead. Since only a single ligand per submission to the server was possible we have carried out docking for only about 300 ligands and manually selected clusters docked in the space relevant to the interface. Finally, the ChemPLP algorithm [38] as implemented in the Gold program [39] was used with the same docking space as in the case of FlexX calculations. This algorithm has been considered as one of the best in most recent benchmark studies [40]. Blind docking in the case of all algorithms was used to check if the binding at the interface is the optimal place for a given ligand. Furthermore, binding to individual proteins (ACE2 receptor and S-protein) has been carried out to investigate the role of ligands (as a binder of binding inhibitor). For the proteins and ligands preparation and visualization, apart from those embedded in the docking programs, Hyperchem [41], Gaussview [42], Chimera [19], and Mercury [43] were used.

### Machine Learning using Random Forest Regressor

All of the 1820 structures were converted from 2D to 3D using RdKit [44] and relaxed at the molecular mechanics level using MMFF94 Merck Force Field [45]. Morgan Fingerprints [27] with a radius of 3 and bit length of 4096 were used as a representation of general structural features of compounds. Calculations were done using Python scripts in the Anaconda environment. Models were built using scikit-learn [46] implementation of Random Forests Regressor with grid optimization of the most important hyperparameters listed in Table 3. The R^2 on the whole training set and Q^2 by five-fold cross-validation were used as metrics for learning and prediction performance, respectively. Each of the considered docking scores modeled delivered a separate hyperparameter set. The final models were validated by leave one out cross-validation procedure.

**Table 3:** Extents of the grid search for best hyperparameters of Random Forests Regressor models.

Hyperparameter name	Values searched
min_samples_split	2, 4, 6, 8, 12, 16, 32, 64
min_samples_leaf	2, 4, 6, 8, 12, 16
max_features	8, 16, 32, 64, 128, 256, 512, 1024, 2048,4 096

**Fig 4:**
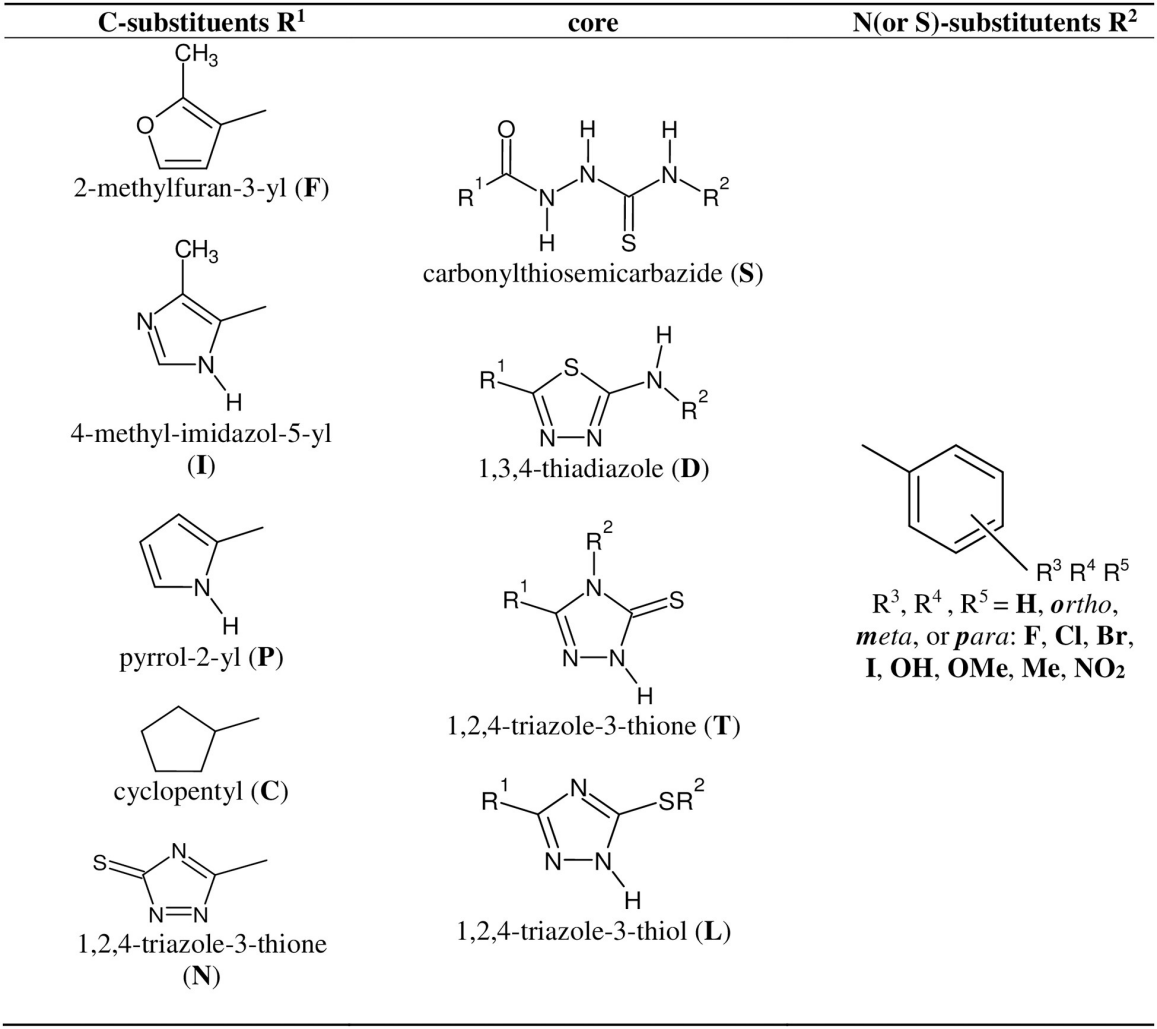
Components of structural fragments of compounds used in current studies.

Once mathematical models were created they were applied to the collection of 597780 structures comprising all variants of R^2^, i.e., all combinations of the phenyl ring decorated with one to five substituents listed in the last column of Fig 4 after removal of structures present in the learning set. This collection was build using our own Python scripts in the Anaconda environment [47].

### ADMET

**Fig 5:**
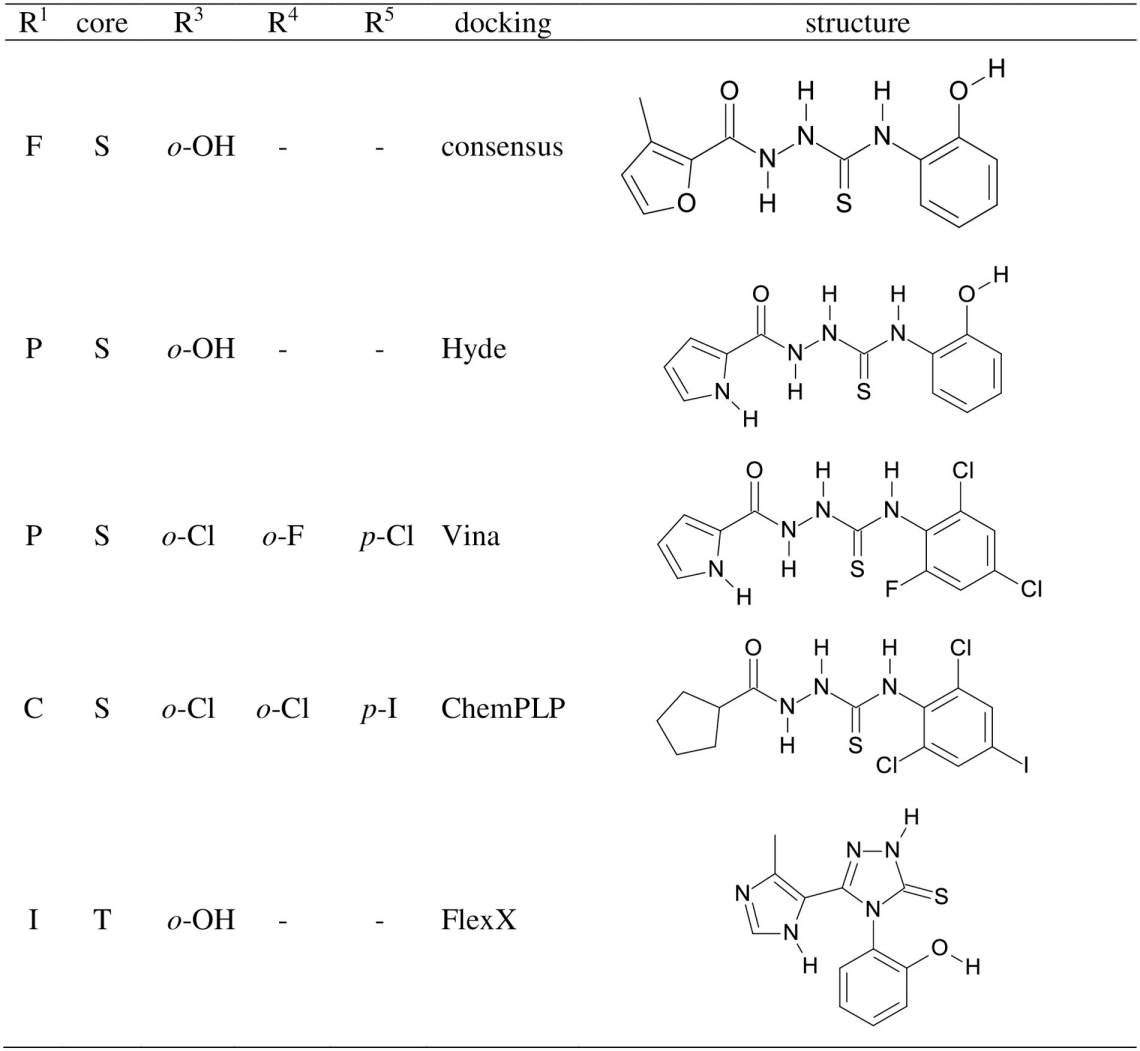
Structure of the molecules with the best scores for individual docking and consensus ranking.

SwissADME program [48] implemented online [49] has been used for the assessment of ADME properties and online implementation [50] of the PreADMET program [51] was used for basic toxicology properties of all 1820 compounds in the training set. For the best results collected in Fig 5 Lipiński’s rules, solubility, and gastrointestinal absorption has been taken from the SwissADME. The toxicity of these compounds to humans (the last five entries reported in Table 2) has been obtained using the online [52] ADMETlab platform [53].
